# Environmental Life Cycle Assessment of Selected Materials—Building Façades in Poland

**DOI:** 10.3390/ma19040807

**Published:** 2026-02-20

**Authors:** Dorota Burchart, Krzysztof Schabowicz

**Affiliations:** 1 Faculty of Transport and Aviation Engineering, Silesian University of Technology, Krasińskiego 8, 40-019 Katowice, Poland; dorota.burchart@polsl.pl; 2 Faculty of Civil Engineering, Wrocław University of Science and Technology, Wybrzeże Wyspiańskiego 27, 50-370 Wroclaw, Poland

**Keywords:** life cycle assessment, carbon footprint, construction sector, ventilated façade, fiber cement board, external thermal insulation composite system

## Abstract

The use of sustainable building materials is becoming increasingly important in order to reduce their environmental impact. This article draws attention to the lack of life cycle assessment (LCA) of building façades, which would take into account national conditions. The aim of the work is to assess the environmental impact of various building façade solutions. The analysis concerned a ventilated façade on an aluminum substructure with a fiber cement board and external thermal insulation composite system (ETICS) with expanded polystyrene (EPS). The assessed façades differed with regard to the used insulation materials. The study aims to select more ecological façades, while at the same time taking into account national conditions, which is important at the stage of designing a building. The study also aims to fill a gap in the existing literature by providing information concerning the environmental analysis of building façades based on real data. Based on a comparative analysis, it was shown that ETICSs with EPS have higher façade-damage category indicators in all impact categories except for eutrophication, human toxicity (carcinogenic and non-carcinogenic), and resource use related to minerals and metals, for which the ventilated façade shows higher values. Additionally, hot-spots for the analyzed façades were also presented. In the case of a ventilated façade, the determinant is the used insulating material, which is mineral wool. In the case of ETICS, it is the finish coat. For the first time in Poland, the LCA of a ventilated façade and ETICS was presented based on real data. The results of this study can be used as the first step of a full cradle-to-grave LCA for buildings.

## 1. Introduction

The implementation of the European Green Deal, increasing requirements from the sustainable development goals, and the transition into a green economy of the European Union have led to transformations in the construction industry. The construction industry is one of the main sources of greenhouse gas (GHG) emissions. It is responsible for approximately 40% of total energy consumption in the European Union, and for 36% of greenhouse gas emissions related to the energy supply of buildings during their use [1,2]. Paper [3] presented the guidelines and initiatives of the European Commission related to environmental issues for the construction industry, for which managing and reducing the carbon footprint is becoming a key challenge. In order to reduce greenhouse gas emissions in the construction industry, it is necessary to conduct research in order to achieve sustainable buildings. To this end, the first step is to recognize and assess the current condition of buildings in terms of their impact on GHG emissions, while at the same time taking into account not only the building operation stage, but also the construction stage. For this purpose, the life cycle assessment (LCA) technique can be used, which is recommended by the European Commission in the new Directive 2023/1791 of the European Parliament of 13 September 2023 on energy efficiency. To date, many environmental impact assessments based on LCA have been performed for the following building materials: concrete [4,5], constructive solutions [6], cementitious materials [7], prefabricated buildings [8], building materials [9], flat roof systems [10], structural materials [11], innovative building materials [12] and building insulation materials [13,14], and residential building walling [15]. Paper [16] shows the approach needed to evaluate the toxicity of building materials using LCA. It was stated that materials such as insulation or paints have a high human health indicator and ecotoxicity impact. A comparative environmental LCA of road asphalt pavement solutions made of artificial aggregates was shown in paper [17]. There are not a lot of publications concerning the LCA of building façades. The façade, as the first element that protects against undesirable external influences, can contribute to the sustainable development of a building by reducing its impact on the environment. Over the last decade, fiber cement boards have become an interesting architectural material, especially those reinforced with more sustainable fibers. Paper [18] presents an innovative model for assessing the durability index of façade boards, and in particular fiber cement boards. There is currently a wide range of renewable materials that can be used to modernize building façades, including wood, cork, recycled materials, and polymer composites. Life cycle assessment is one of the decision criteria of Multi-Attribute Decision-Making applications in façade design [19,20].

Based on a state-of-the-art review, it was found that several LCA studies have already been carried out regarding the modernization of building façades. Paper [21] presents the potential of abaca fibers to contribute to the circular economy and carbon footprint reducing. Paper [22] shows a LCA for different wall assemblies made of stabilized rammed earth reinforced with natural fibers. The carbon footprint of an insulating material was presented in accordance with the life cycle approach [23]. Other authors focused on the analysis of the environmental impact of the production of lime mortar as a decorative mineral plaster for ETICSs. In paper [24], there are also the results of the analysis of the Multi-Active façade system with ETICS and EPS. There was a lack of research on the analysis of the life cycle of ventilated façades, which would take into account actual data concerning the production of a ventilated façade.

Performing LCA analysis is not yet required in accordance with Polish legal acts; however, taking into account changes in the Directive, as well as the Renovation Wave strategy, the LCA technique becomes an important tool that can help in the analysis of the environmental impact of a building’s life cycle. Only a life cycle approach will fully assess and improve the environmental performance of buildings. Until now, there has been no developed LCA methodology for buildings in Poland that covers national conditions. Developing such a methodology and determining the limit indicators for greenhouse gas emissions in a building’s life cycle is a complex process. For these reasons, LCA studies covering individual building elements are important. Therefore, this work focused on the analysis of one of the most important elements of a building, which is the façade system.

The aim of this article is to perform an LCA analysis for a building’s façade based on actual data, while at the same time taking into account the life cycle of the individual materials and elements of the façade. The tests were performed for Polish conditions, which is associated with the fact that insulating materials of greater thickness had to be used in order to meet the thermal insulation requirements and other requirements related to energy saving, A comparative LCA analysis of a ventilated façade with the use of ETICS was performed. In turn, ETICS with EPS was used for comparative analyses, which is due to the fact that it is a system used in buildings in Poland (it has an attractive price). The conducted analysis allowed for the specifying of which system is more ecological, and also showed the hot-spots in each of the façade’s systems. Performing an LCA analysis is the first step towards identifying changes and innovations in buildings by relating these changes and innovations to all the factors and materials in the building’s life cycle. In the article, the assessment of the life cycle of a ventilated fiber cement board and ETICS was described for the first time in Poland.

## 2. Materials and Methods

The assessment of the potential impact of a ventilated fiber cement board and ETICS was performed using the life cycle assessment method following the international ISO standards [25,26]. The LCA study includes a definition of its goal and scope, life cycle inventory (LCI), life cycle impact assessment (LCIA), and ends with an interpretation of the results and recommendations [27,28].

### 2.1. Goal and Scope of the LCA Analysis

The aim of the research was to perform an LCA analysis of building façades that are used in Poland. Both façades were modeled with identical system boundaries, functional unit, harmonized data-collection protocols and the same life cycle impact assessment method. The life cycle assessment was performed using SimaPro Craft 10.3 software with the Ecoinvent database version 3.9 [29]. The “cut-off” system model was adopted, which is consistent with attributional LCA and aligned with the Environmental Footprint (EF 3.1) methodology recommended by the European Commission. The study follows an attributional, process-based approach. Primary data describing façade components and installation processes were directly linked to background processes from Ecoinvent 3.9.

In the construction industry, it is helpful to use EN 15804:2012+A2:2019 [30] and EN 15978:2011 [31] standards for life cycle analysis [32]. For the purpose of the analysis, the rules (PCR—Product Category Rules) for thermal insulation materials were taken as a reference. This is due to the fact that they do not exist specifically for ETICS and ventilated façade systems. For comparative purposes, all analyses were referred to the same functional unit. The used functional unit (FU) was 1 m^2^ of a building façade, as in studies [20,33,34]. Based on the analysis of previous LCA studies of various façade systems, the life cycle of a façade system was considered to be 50 years [21,22,35,36].

### 2.2. The Building Façades’ Definition and System Boundary

**Figure 1:**
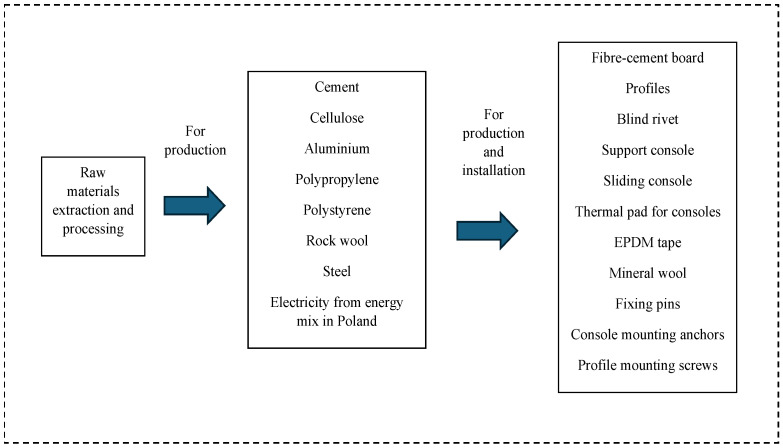
System boundary of the ventilated façade system.

**Figure 2:**
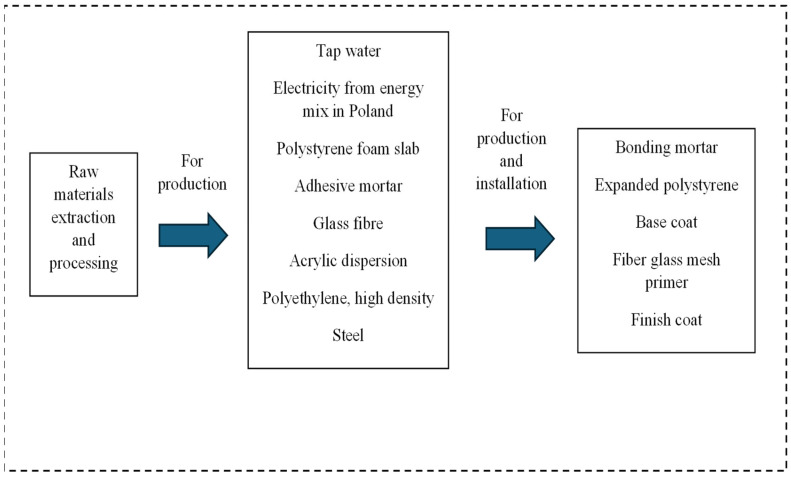
System boundary of the ETICS.

The aim of this research is to assess the potential environmental impact of two building façade solutions that are used in Polish conditions. The objects of the research included a ventilated façade with a fiber cement board, and ETICS with EPS. The system’s boundary was defined, and sets of inputs and outputs were identified as elements of individual system phases. The system boundaries of the study are compliant with ISO 14040:2006 [25] and ISO 14044:2006 [26] standards. The system’s boundary covers the scope of processes from cradle to gate: resources used and their respective life cycles, all needed materials and energy production and individual façades’ production and installation. The system’s boundaries for individual façades are shown in Figure 1 for the ventilated façade and in Figure 2 for ETICS.

In LCA analyses, in order to demonstrate the reliability of the analysis results, it is important to present the assumptions and limitations of the analysis. Outside of system boundaries are transport and “end of life” stage. Transport of materials is not included due to the difficulty of determining distance, while the “end of life” stage is not included due to the lack of data on the possibility of taking into account the recycling of individual materials after being used for many years. Such cut-off criteria echo an earlier paper regarding LCA analyses for façade systems in other countries. An additional argument for limiting the analyses to the stage of production and installation of the façade system is the purpose and scope of the work, which focuses on comparing the materials used in the production of individual façades. The cradle-to-gate LCA of a building’s façade covers both the raw materials extraction, material and energy production process and the installation stage of a building’s façade.

An example of a façade that uses fiber cement boards, a schematic cross-section of the ventilated facade system and a schematic cross-section of the ETICS is available in the Supplementary Materials [Figures S1–S3].

The first type of façade analyzed is a ventilated façade with a fiber cement board.

There is no Polish standard for ventilated façades that considers a whole system. For a given type of ventilated façade that is built on a building, technical approval or technical documentation prepared by the designer is required in order to approve it for individual use [37]. When preparing technical documentation, a designer selects the parameters of individual system components and, on this basis, determines the properties of the entire façade, which must comply with the technical conditions [38,39]. Each component of the façade should be provided with appropriate documents, i.e., a declaration of performance and a CE marking. Ventilated façades are a set of construction products that create an entire system, and should be considered as a whole. Based on the guidelines for European technical approvals, ETAG 034, a technical approval—currently a national technical assessment—was developed for a ventilated façade. These guidelines define the scope of test methods and requirements for a complete façade system. In Poland, technical documents of this type are developed and published by the Building Research Institute [33]. In terms of widespread use, fiber cement boards are the leader among façade claddings and therefore in this study the life cycle assessment of ventilated façade systems with fiber cement boards was performed.

These boards are produced in various varieties. Gray boards are commonly available, but they can be colored according to the color palette offered by the manufacturers. They can also be painted, coated with materials that leave patterns, or can be made of certain materials, e.g., wooden planks. Ventilated façades made of fiber cement boards are very popular in Scandinavia, Russia and North America, although they differ significantly in terms of the used raw materials. In Poland, the use of fiber cement boards is also growing, which is related to their good fire protection and strength parameters. When assessing building façade systems, the environmental aspect is becoming increasingly important. It takes into account the life cycle, starting from the extraction of raw materials, through the production of materials, to the production process of the façade itself.

A ventilated façade is a set of appropriately selected elements that create a complete façade system. This system consists of a substructure called a grid, thermal insulation, a ventilation gap, and the façade’s cladding made of fiber cement boards.

An important element of the façade system is the ventilation gap in which air flows between the thermal insulation and the façade’s cladding. The flowing air carries away the condensate and moisture that has accumulated in the wall outside the partition. The substructure creates a skeleton, which is used to attach the cladding at a certain distance from the structural wall (taking into account the thickness of the thermal insulation and ventilation gap). The ventilation gap thickness may vary, depending on, among other things, the faults of the wall structure elements, the thickness of the thermal insulation material, and the adopted architectural conditions. The substructure can be made of aluminum, wood, steel or composite material. Thermal insulation is responsible for large part of thermal performance, but only to a minor extent on acoustics. It should also have high vapor permeability and be protected from the outside with a veil or membrane that protects against wind and moisture. The last element of the façade system is the façade’s cladding, which primarily gives the facility its appropriate appearance. It must also ensure the drainage of rainwater and protect the thermal insulation against external environmental and exceptional factors. Façade cladding can be made of various materials, and is available in various colors and formats. One of the more popular solutions used by architects and investors are façade claddings made of fiber cement boards, which were selected for analysis in this study. The growing interest in these types of building façades is also influenced by the perceived shortcomings of the very popular façades that were used in the past and which constitute a complex thermal insulation system with ETICS plasters.

The system boundary of the ventilated façade is depicted in Figure 1.

The system boundary includes all raw materials, materials and energy production and the ventilated façade system production and installation.

The analysis included all the elements of the ventilated façade system, but without the structural wall. The thickness of the fiber cement boards was 8 mm, and the dimensions of the board were 1250 × 3050 mm. The bulk density of the basic board was 15 kg/m^2^, and its average bending strength was 24 MPa. Fiber cement boards are non-flammable and have a fire resistance class of at least A2.

The growing popularity of ventilated façades is due to the possibility of using various cladding materials, which are tailored to the requirements for modern façades. This makes it possible to use ventilated façades in various conditions, regardless of the environment and fire requirements, and also with the possibility of obtaining appropriate strength. The structural element of a ventilated façade is the substructure (grid). In the analyzed case, it was made of aluminum.

This solution is currently the most popular due to its large range, simple installation, the possible joining of the façade’s cladding (both visible and invisible), and the possibility of using cladding from various materials. Therefore, this solution was used in this research. The grate consists of vertical aluminum profiles and mounting brackets, which are called consoles. The vertical elements of the grid are made of T-sections. The width of the profile provides adequate support for two adjacent façade panels, along with an 8–12 mm wide expansion gap between the panels. Angle bars were used as intermediate support for the panels, and were used in the corners of the façade. The vertical profiles were mounted on the structural wall using consoles made of unequal angle bars. The consoles’ reach depends on the distance between the structural wall and the cladding. This distance is necessary to assemble thermal insulation and to provide a ventilation gap of 20 to 50 mm according to ETAG 034 guidelines. Typical console reach is 200–300 mm. The consoles are equipped with special handles (clips that facilitate the installation of aluminum profiles) and a stiffening bracket, which is necessary for greater overhang. The length of the vertical profiles was limited to approximately 3.0 m due to the high thermal expansion of aluminum. Vertical profiles were connected to the consoles using rivets, while taking into account the principle of one fixed fixing point. The remaining joints were slidable, which enables displacements that result from temperature changes to be compensated. It should be remembered that additional stiffening of the grid structure cannot be introduced when designing and assembling the substructure. EPDM spacers were used to enable free movement of the profiles and the façade’s panel against each other. The spacer additionally covers the bright aluminum structure that is visible in the joint. Connectors, made of the same material as the joined element of the structure, were used to connect aluminum elements. If the connectors are selected incorrectly, electrochemical corrosion may occur. This phenomenon should be taken into account in aggressive environments or, for example, in seaside conditions. The fixing of the façade cladding should ensure that the cladding does not fall off in the event of a fire, and it should not be below the required fire resistance class of the external wall for the appropriate fire class of the building. The aluminum substructure meets the necessary requirements in terms of fire resistance, which was confirmed in tests commissioned by the system’s manufacturers, including the tests presented in paper [37].

The second type of analyzed façade system was the external thermal insulation system for buildings (ETICS), a complex system of the insulating external walls of a building, which was previously called the seamless thermal insulation system, and even earlier, the light-wet method. The essence of this method comes down to making layers of cooperating and compatible materials on a properly prepared surface (wall), which include thermal insulation and a finished façade layer.

Figure 2 shows the system boundary for the ETICS analysis. As shown in Figure 2, the system boundary includes all raw materials, materials and energy production and ETICS production and installation. This system consists of basic ingredients (adhesive mortar, thermal insulation, mechanical connectors (pins), a reinforcing layer, a finish layer) and additional components (materials for finishing details: skirting boards, protective angle bars, expansion profiles, sealing materials, and other necessary accessories).

Each material used in this building façade system has a different function: thermal insulation ensures adequate thermal insulation and is made of expanded polystyrene board (EPS); adhesive mortar and mechanical connectors (pins) ensure an adequate structural stability of the system; the reinforcing layer (a layer of mortar with an embedded mesh, e.g., made of fiberglass) ensures resistance to damage (e.g., due to impacts) and constitutes a base for the finishing façade layer; and the finishing façade layer (plaster, façade tiles) protects the system layers against weather conditions and aging, and also constitutes a decorative layer.

The selection of a façade system depends on several factors. Generally speaking, it concerns structural safety, fire safety, operational safety, acoustic protection, thermal protection, energy saving, and health issues. Therefore, the approach that states that design is only limited to determining the thickness of the thermal insulation layer to meet the thermal protection requirements and the method of fastening (gluing, doweling, depending on the type of thermal insulation and wear layer) should be considered incorrect. The starting point should be an analysis of the investment’s efficiency based on the analysis of operating costs and costs related to the investment. Afterwards, the specificity of the insulated building (the material from which the external walls are made, the shape of the building, the investor’s esthetic requirements), its purpose and location, and the financial resources available to the investor need to be analyzed. Fire and acoustic protection requirements must also be taken into account. No less important is the analysis of the correctness of the adopted solution in terms of the requirements of building physics.

The main methodological limitations of this study include the following:

- System boundary is from cradle to gate, excluding transport and end-of-life stages.
- Partial reliance on secondary datasets for certain materials where detailed industrial data were unavailable.
- High uncertainty associated with selected impact categories, particularly water use and human toxicity, due to characterization model sensitivity.

### 2.3. Life Cycle Inventory

Due to the fact that this is the first LCA study for building façades in Poland, an important element was the identification of quantitative data for the needs of analysis, which comes from the authors’ own studies of façades and data from the producers of individual materials. It should be emphasized that the performed analyses are innovative. Identified inventory data for the analysis of building façade systems, which take into account national conditions, was presented for the first time. The main sources of the data are manufacturing companies.

The primary data used in this study refer to the year 2024 and are representative of current industrial practice in Poland. Geographical representativeness corresponds to production processes and façade installations carried out within Poland. In the case of the absence of more detailed data on some materials, input and output data were determined based on literature data and the Ecoinvent database.

In the case of the analysis of the building’s ventilated façade, all the materials used to develop the ventilated façade with fiber cement board (used as a ventilation cladding) were taken into account. Table 1 presents the inventoried data for the creation of a ventilated façade. All the data were identified and inventoried based on the authors’ own research. The data were related to the functional unit.

The LCA analysis included a ventilated façade with an aluminum substructure. Fiber cement boards with dimensions of 1250 × 3050 × 8 mm were mechanically attached to aluminum profiles with rivets. Thermal insulation was made of mineral wool, which is a thermal insulation material with a low thermal conductivity coefficient. Rock wool was included in this analysis. For the analysis, 14.58 kg/FU of mineral wool with a veil, which was 15 cm thick and had a density of 90 kg/m^3^, was considered. Aluminum profiles and rivets were used for the ventilated façade. The supporting console and the sliding console were also made of aluminum. The material used to make the EPDM tape (Firestone Building Products, Zaventem, Belgium) was Ethylene-propylene-Dien-Monomer. PVC was used to produce the thermal pad for the consoles. The console mounting anchors and profile mounting screws were made of steel.

In Poland, when compared to countries such as Portugal, a thicker layer of wool should be used due to climatic conditions and in order to meet the thermal insulation requirements and other requirements related to energy-saving practices specified in the Regulation of the Minister of Infrastructure, regarding the technical conditions to be met by buildings and their locations [40]. The raw materials shown in Table 2 were used to produce fiber cement boards [41].

Detailed requirements for cement, limestone, cellulose fibers and PVA for the production of fiber cement boards were presented in the authors’ previous works [33,35].

Regarding the analysis of ETICS with EPS, all the materials used to produce the building façade were taken into account. In the case of the data for the analysis of ETICs, in addition to actual data that takes into account national conditions, secondary data based on literature data were also used. The following components were used to analyze the ETICS: EPS, bonding mortar, base coat, fiberglass mesh, primer, finish coat. The amount of polystyrene foam slab was adjusted to comply with national conditions. Its thickness was assumed to be 15 cm in order to meet the thermal insulation requirements and other requirements related to energy saving specified in the Regulation of the Minister of Infrastructure on the technical conditions to be met by buildings and their locations. The calculations resulted in an amount of 2.7 kg of polystyrene foam slab per 1 m^2^ of façade.

Table 3 presents the inventoried data for the creation of ETICS with EPS, based on the authors’ own research. The data analysis included both research-based data and literature-based data. All the data were related to the functional unit.

The Polish electricity mix was modeled follow “Energy Transition in Poland. 2025 Edition” [42]. The use of the Polish electricity mix ensures geographical consistency of the modeling framework and reflects national energy conditions relevant for façade production and installation processes.

The exclusion of these elements is due to difficulties in quantifying and inventorying the data. It was also intended to give greater importance to the remaining elements of each system, i.e., the thermal insulation and external cladding.

### 2.4. Life Cycle Impact Assessment

An important stage of the work was the identification and inventory of data regarding the construction of the façade for which the LCA analyzes were performed. The next element of the study involved the assessment of the associated environmental loads, both direct and indirect, and the identification of the main sources of the environmental impact of façades. A quantitative assessment of potential environmental burdens related to the raw materials and the materials used to construct a building’s façade was performed. LCA was performed in accordance with ISO 14044:2006 [26].

A cradle-to-gate inventory combined primary data of the chosen building’s façades installation with secondary datasets from Ecoinvent 3.9. Sixteen midpoint damage categories were calculated with the European Commission’s Environmental Footprint 3.1 (EF 3.1) method [43] and normalized per functional unit. All sixteen impact categories recommended by the European Commission were included: climate change (CC), ozone depletion (OD), human toxicity—cancer (HT-c), human toxicity—non-cancer (HT-nc), particulate matter (PM), ionizing radiation (IR), photochemical ozone formation (POF), acidification (AP), eutrophication terrestrial (ET), eutrophication freshwater (EF), eutrophication marine (EM), ecotoxicity freshwater (EFT), land use (LU), water use (WU), resource use—minerals and metals (RMM), and resource use—fossils (RFs) [28]. The normalization step was applied according to the respective factors provided by the European Commission [43] and no weighting factors have been applied.

## 3. Results and Discussion

The results from the comparative analysis of the environmental impact categories of the used types of façades in Poland are presented in Table 4 (characterization stage) and damage categories are presented in Table 5 (damage assessment stage).

Based on the LCA analyses performed, it was shown that carbon footprint (which is the most important aspect according to the European Commission guidelines) was higher for the ETICS with EPS than for the ventilated façade with rock wool. Based on the results of analyses, it was also found that it is not possible to clearly determine which façade is more ecological when considering all the impact categories. This is because it depends on the impact category taken into account. The ventilated façade exhibits higher impact indicators in the following damage categories: eutrophication, human toxicity (both carcinogenic and non-carcinogenic), as well as resource use related to minerals and metals. In all remaining impact categories, the environmental burden is higher for the ETICS.

Impact categories for the compared façade systems are shown in Table 4. Based on the performed LCA analyses for the two façade systems, as summarized in Table 5, it was concluded that the ETICS exhibits higher damage category indicators in all impact categories except for eutrophication (freshwater and terrestrial), human toxicity (carcinogenic and non-carcinogenic), and resource use related to minerals and metals, for which the ventilated façade shows higher values. Table 6 and Table 7 present the determinants of life cycle assessment for ventilated façades, taking into account damage category. Table 8 and Table 9 present the share of individual elements in the life cycle assessment of the evaluated damage categories for the ventilated façade system and ETICS.

Elements in the life cycle assessment for ventilated facades and ETICS in the characterization stage according to the EF method and share of individual elements in LCA of the evaluated impact categories for these facades are available in the Supplementary Materials [Tables S1–S4].

The impact category “acidification” shows a higher environmental index for ETICS than for ventilated façades. In the case of ETICS, more than 60% of the acidification impact is associated with the finish coat. For ventilated façades, mineral wool contributes the most to this category, accounting for nearly 60% of the total impact of acidification.

For the impact category of climate change, ETICS represents the less favorable solution, whereas the ventilated façade exhibits lower environmental impacts. In the subcategories “climate change—biogenic” and “climate change—fossil,” ETICS also demonstrates higher values. In contrast, for “climate change—land use,” the indicator is higher for the ventilated façade. For the climate change impact category—land use and LU change, more than 70% of the environmental burden associated with the ventilated façade is attributable to the production of aluminum profiles, which largely accounts for the elevated contribution observed for this system component.

In the case of ETICS, the highest contribution to the climate change indicator is associated with the finish coat, amounting to 19.86 kg CO_2_-eq, followed by the EPS layer, which accounts for 11.61 kg CO_2_-eq. In contrast, for the ventilated façade, the largest climate change impact is linked to the production of mineral wool (16.63 kg CO_2_-eq), followed by the production of the fiber cement board (8.10 kg CO_2_-eq) and aluminum profiles (7.14 kg CO_2_-eq).

For all ecotoxicity indicators—except for “ecotoxicity freshwater, organic P1”—the ETICS shows higher ecotoxicity values compared with the ventilated façade. With respect to the contribution of individual material components, in the case of the ventilated façade, the highest share in “ecotoxicity freshwater, organic P1” originates from aluminum profiles, accounting for more than 50% of the total impact. Mineral wool represents nearly 35% of the impact in this category. For the indicator “ecotoxicity—organic P2,” mineral wool is the dominant contributor, representing 48% of the overall impact, while aluminum profiles account for approximately 30%. For the remaining ecotoxicity categories, the highest impact is likewise associated with the production of mineral wool. In contrast, for the ETICS, the production of the finish coat constitutes the largest contribution to the ecotoxicity indicators.

For the impact category “particulate matter,” the ETICS demonstrates a higher indicator compared with the ventilated façade. In the ventilated façade, the largest contribution to this category—53.9%—is associated with the production of mineral wool, followed by aluminum profiles, which account for approximately 25%. In the case of ETICS, the dominant contribution to particulate-matter emissions originates from the finish coat (over 53%), while nearly 20% is attributed to EPS.

Similarly to the particulate-matter impact category, the marine eutrophication indicator is also higher for the ETICS compared with the ventilated façade.

For the impact category of freshwater eutrophication, the ventilated façade shows a higher indicator compared with the ETICS. In the case of the ventilated façade, this impact is primarily associated with the production of aluminum profiles, whereas for ETICS it is mainly linked to the production of the finish coat.

For the terrestrial eutrophication impact category, the indicator is also higher for the ventilated façade compared with the ETICS. In the ventilated façade, more than 55% of the impact is associated with the production of mineral wool, while approximately 21% results from the production of aluminum profiles. In the case of ETICS, terrestrial eutrophication is predominantly driven by the finish coat, which accounts for over 51% of the impact, followed by EPS with a contribution of nearly 20%.

The analysis also demonstrated that, for the impact category “human toxicity,” the indicators for all subcategories—both cancer and non-cancer—are higher for the ventilated façade than for the ETICS.

Within the human-toxicity impact category, it was demonstrated that, for the ventilated façade system, the major determinants of the total impact are the fiber cement board, mineral wool, and aluminum profiles. The fiber cement board contributes more than 80% to the subcategory “human toxicity, cancer inorganic” and more than 90% to “human toxicity, non-cancer inorganic.” Mineral wool accounts for over 80% of the impact in the subcategory “human toxicity, cancer organic,” whereas aluminum profiles contribute more than 55% to “human toxicity, non-cancer organic.”

In the case of the ETICS, the finish coat constitutes the dominant contributor to the human-toxicity impact category, accounting for between 40% and 60% of the total impact, depending on the specific subcategory.

For the remaining impact categories—including ionizing radiation, land use, ozone depletion, photochemical ozone formation, resource use (fossils), and water use—higher indicators were observed for the ETICS. In all of these categories, the elevated values for ETICS are primarily driven by the production of the finish coat, which contributes between 45% and 60%, depending on the specific impact category. In the case of the ventilated façade, the impacts in these remaining categories are predominantly associated with the production of mineral wool.

Only in the case of the impact category “resource use—minerals and metals” was a higher indicator observed for the ventilated façade compared with the ETICS. This result is primarily driven by the extensive use of aluminum in the production of aluminum profiles, which accounts for more than 75% of the impact in this category for the ventilated façade. In contrast, for the ETICS, this impact category is mainly influenced by the finish coat, contributing approximately 65%. Uncertainty analysis for the ventilated façade and uncertainty analysis for ETICS are available in the Supplementary Materials [Tables S5 and S6].

One of the limitations of LCA is the uncertainty problem. These uncertainties will directly affect the correctness and reliability of the LCA research conclusions. The Monte Carlo method, as statistical simulation method, is a method of numerical calculation guided by probability statistical theory.

Uncertainty analysis was performed for the ventilated façade system. The Monte Carlo uncertainty analysis provides a quantitative assessment of the robustness and variability of the impact characterization results for the ventilated façade system. Across the evaluated impact categories, the magnitude of uncertainty varies substantially, reflecting differences in data quality, model structure, and the inherent variability of underlying life cycle processes. Overall, several impact categories exhibit low relative uncertainty, as indicated by modest standard deviations (SD), narrow 95% confidence intervals, and low coefficients of variation (CV). Categories such as photochemical ozone formation (CV ≈ 6.99%), climate change (CV ≈ 7.10%), and resource use, fossils (CV ≈ 7.09%) demonstrate particularly stable outcomes. Their narrow confidence intervals and low standard errors of the mean (SEM) suggest that the underlying inventory data are consistent and that the model outputs are not highly sensitive to parameter variability. These categories can therefore be interpreted with a high degree of confidence.

In contrast, several categories show substantial uncertainty, with extremely high CV values and wide confidence intervals. The most pronounced example is water use, which displays a CV exceeding 13.500% and an SD two orders of magnitude larger than the mean. Similar issues are observed in Human toxicity, non-cancer and its subcategories, where CV values exceed several thousand percent, reflecting very high sensitivity to parameter uncertainty and potentially low reliability of the available toxicity characterization data.

Uncertainty analyses were also performed for ETICS.

The Monte Carlo uncertainty analysis conducted for the ETICS reveals substantial variation in the robustness of impact characterization results across environmental categories. The magnitude of uncertainty differs markedly between categories, reflecting the heterogeneity of underlying life cycle inventory data, the sensitivity of specific impact pathways, and the methodological limitations inherent to certain characterization models. Several impact categories exhibit relatively low to moderate uncertainty, as indicated by modest standard deviations (SD), narrow 95% confidence intervals, and coefficients of variation (CV) below approximately 15%. This group includes climate change (CV ≈ 9.85%), Climate change—fossil (CV ≈ 9.84%), eutrophication, marine (CV ≈ 10.77%), eutrophication, terrestrial (CV ≈ 10.74%), and photochemical ozone formation (CV ≈ 12.43%). These results suggest that the underlying inventory data for these categories are comparatively consistent and that the model outputs are not highly sensitive to parameter variability. Consequently, these categories can be interpreted with a higher degree of confidence, and the results are considered robust for comparative or decision-support purposes.

The highest uncertainty is observed in categories with very large CV values, wide confidence intervals, and substantial divergence between mean and median values. Notably, eutrophication, freshwater (CV ≈ 47.27%), human toxicity, non-cancer—inorganics (CV ≈ 47.54%), resource use, minerals and metals (CV ≈ 50.66%), and especially water use (CV ≈ 604%) exhibit extreme variability. In the case of water use, the SD (206.17 m^3^) is several times larger than the mean (34.12 m^3^), and the confidence interval spans from negative to highly positive values, indicating a highly skewed or unstable distribution. Such patterns typically reflect poorly constrained inventory data, the presence of outlier processes with disproportionately large contributions, or methodological limitations in water scarcity characterization models. These categories should therefore be interpreted with significant caution, and their results may not be reliable for comparative decision-making without further refinement of input data.

Based on the performed LCA it was shown that the ETICS exhibits higher damage-category indicators in all impact categories except for eutrophication, human toxicity (carcinogenic and non-carcinogenic), and resource use related to minerals and metals, for which the ventilated façade shows higher values.

The LCA analysis showed that the main determinant of carbon footprint in the case of ventilated façades is the production of mineral wool. Other significant carbon footprint factors include fiber cement board with lime (where the impact of the board is mainly related to the production of cement) and production of aluminum profiles. It has also been shown that thermal insulation consisting of mineral wool is the main source of influence on acidification, human toxicity-cancer-organics and fossil resource scarcity. The production of fiber cement board was also found to be the factor with the highest impact on human carcinogenic toxicity (inorganics). The impact is primarily related to the production of cement. Clinker production is the main source of negative environmental impact in cement production technology. Human toxicity-cancer-inorganics is caused by the emission of heavy metals from the clinker production process. Human carcinogenic toxicity is mainly connected to the chromium burden generated from transportation, petroleum refinery operation, and coal washing in the clinker production process. Another important factor that causes the highest environmental impacts are aluminum profiles. The profiles are the main determinants of impacts in the impact categories: mineral and metal resource scarcity, ecotoxicity—organics, eutrophication, and climate change, especially a subcategory related to land use. The negative impact of the profiles is primarily related to the aluminum production process, which is very energy intensive.

In the case of the ETICS with EPS assessment, it was shown that the main determinant of the environmental assessment is the finish coat, and especially the use of acrylic dispersion for the finish coat. Other factors that significantly influence the damage categories include the production of EPS and the adhesive mortar that is used in the production of the bonding mortar and base coat. It has been shown that the environmental impact of the ETICS is determined primarily by the acrylic façade paint (acrylic dispersion), and to a lesser extent it depends on the thermal insulation material, i.e., EPS. For this reason, silicone or silicate façade paints are being used more and more often. However, such solutions require further LCAs.

## 4. Conclusions

In accordance with the guidelines of the European Commission, actions should be taken to reduce the consumption of raw materials and energy, as well as to reduce greenhouse gas emissions during the life cycle of buildings. Due to the fact that the construction industry is responsible for a high consumption of energy and natural resources, a big production of waste, and the fact that it is one of the main sources of greenhouse gas emissions, it is necessary to look for solutions and technologies that will reduce the negative impact of this sector on the environment. This is why research such as this one is important, the aim of which is to demonstrate the environmental impact of the materials used in building façade systems.

The LCA life cycle assessment technique is considered a key method for quantifying and assessing the environmental impact of buildings. The conducted research and the obtained results of LCA analyzes for building façade systems can help decision-makers determine the stages of a façade’s life cycle which have the highest impact on the environment. The LCA of a façade that uses insulating materials that are very popular in Poland was conducted during the research.

The article presents the results of the life cycle analysis of individual materials and façade elements based on actual data for Polish conditions. This means that insulating materials of relevant thickness had to be used to meet the requirements of thermal insulation and other requirements related to energy saving, which are specified in the Regulation of the Minister of Infrastructure on the technical conditions to be met by buildings and their locations.

It was shown that the factors that have the greatest impact on the environment, when taking into account the life cycle assessment of the ventilated façade, are mineral wool, fiber cement board and profiles. However, in the case of the ETICS with EPS assessment, it was shown that the main determinant of the environmental assessment is the finish coat, whereas the impact of the thermal insulation material, i.e., EPS, is lower. In the case of the life cycle assessment of the ventilated façade, it was shown that the most significant impact is the thermal insulation material, which is mineral wool.

The Monte Carlo uncertainty analysis demonstrated that certain impact categories, including climate change, fossil resource use, marine eutrophication, and photochemical ozone formation, show relatively low coefficients of variation and narrow confidence intervals. Differences observed in these categories between façade systems can therefore be considered robust. In contrast, categories such as water use, resource use (minerals and metals), and selected human toxicity indicators exhibit high variability and wide confidence intervals. For these categories, the comparative differences should be interpreted with caution, as the uncertainty ranges partially overlap and reflect limitations of available inventory data and characterization models. Consequently, while the general trend indicates higher environmental burdens for ETICS in most categories under the baseline configuration, not all differences are statistically robust across all impact categories.

The results indicate that, while ETICS shows higher impact values in most categories under the baseline configuration, the statistical significance of these differences varies across impact categories. In some cases, confidence intervals partially overlap, suggesting limited statistical robustness. However, the comparative trend remains consistent across the tested parametric variations.

Performing an LCA analysis is the first step towards identifying changes and innovations in buildings by relating them to all the factors and materials in a building’s life cycle.

The article presents, for the first time in Poland, a life cycle assessment of both a ventilated façade made of fiber cement board and the ETICS.

Subsequent research will present new low-energy methods of producing insulating materials, and at the same time will take into account the climatic conditions of individual countries. The results of this study offer a comprehensive environmental analysis of Polish building façade systems, and can be used as the first step in performing a holistic LCA of a building from cradle to grave that includes all the phases of the life cycle.

The authors of the article are currently working on possible alternatives to the determinant contributions and will present the results of their research in subsequent publications.

## Figures and Tables

**Table 1:** Inventory of materials of ventilated façade system.

Components	Inputs	Amount	Unit
Fiber cement board	Fiber cement	15.273	kg
Profile T T120/70/2	Aluminum	1.776	kg
Profile L L150/70/2	Aluminum	0.778	kg
Blind rivet 4.0 × 20 K14	Aluminum	0.031	kg
EPDM tape 60 mm	Etylen-propylen-Dien-Monomer	0.016	kg
EPDM tape 120 mm	Etylen-propylen-Dien-Monomer	0.281	kg
Support console	Aluminum	0.289	kg
Sliding console	Aluminum	0.397	kg
Thermal pad for consoles	PVC	0.031	kg
Mineral wool	Mineral wool	14.580	kg
Fixing pins	Polipropylen	0.313	kg
Console mounting anchors	Steel	0.413	kg
Profile mounting screws	Steel	0.007	kg
Installation	Electricity from energy mix in Poland	0.08	kWh

**Table 2:** Comparison of testing [41].

Raw Material	Approximate Contents ^a^
Cement	60%
Cellulose (dry)	8%
PVA	2%
Lime	30%
Total	100%
Additives and admixtures	/
Superplasticizer	0.1 dm^3^/t
Didecyldimethylammonium chloride (DDAC) or bromide (DDAB)	
Perlite	1 kg/t
Mica	
Microsphere	
Antifoaming agent	0.26 dm^3^/t

^a^—Concentrations dm^3^/t and kg/t refer to the content in the finished product.

**Table 3:** Inventory of materials of ETICS with EPS.

Components	Inputs	Amount	Unit
Bonding mortar	Adhesive mortar	4.500	kg
	Tap water	1.350	kg
	Electricity from energy mix in Poland	0.016	kWh
EPS	Polystyrene foam slab	2.7	kg
Base coat	Adhesive mortar	4.500	kg
	Tap water	1.350	kg
	Electricity from energy mix in Poland	0.016	kWh
Fiberglass mesh	Glass fiber	0.330	kg
Primer	Acrylic dispersion	0.440	kg
	Polyethylene, high density	0.016	kg
	Tap water	1.944	kg
Finish coat	Polyethylene	0.290	kg
	Steel	0.012	kg
	Acrylic dispersion	8.000	kg

**Table 4:** Comparative LCA for building façades in Poland: Impact category, characterization stage.

Impact Category	Unit	Ventilated Fascade with Rock Wool	ETICS with EPS-PL
Acidification	mol H+ eq	0.2776	0.4375
Climate change	kg CO_2_ eq	36.8156	42.5229
Climate change—Biogenic	kg CO_2_ eq	0.0617	0.1255
Climate change—Fossil	kg CO_2_ eq	36.6420	42.3688
Climate change—Land use and LU change	kg CO_2_ eq	0.1118	0.0285
Ecotoxicity, freshwater—part 1	CTUe	85.3324	161.5034
Ecotoxicity, freshwater—part 2	CTUe	13.1038	25.0509
Ecotoxicity, freshwater—inorganics	CTUe	80.8024	160.7112
Ecotoxicity, freshwater—organics—p.1	CTUe	10.9023	3.8792
Ecotoxicity, freshwater—organics—p.2	CTUe	6.7314	21.9640
Particulate matter	disease inc.	2.32 × 10^−6^	2.54 × 10^−6^
Eutrophication, marine	kg N eq	0.0368	0.0417
Eutrophication, freshwater	kg P eq	0.0176	0.0157
Eutrophication, terrestrial	mol N eq	0.4996	0.4173
Human toxicity, cancer	CTUh	6.47 × 10^−8^	5.78 × 10^−9^
Human toxicity, cancer—inorganics	CTUh	1.11 × 10^−9^	3.50 × 10^−15^
Human toxicity, cancer—organics	CTUh	6.36 × 10^−8^	5.78 × 10^−9^
Human toxicity, non-cancer	CTUh	1.07 × 10^−7^	2.93 × 10^−8^
Human toxicity, non-cancer—inorganics	CTUh	6.38 × 10^−8^	1.61 × 10^−9^
Human toxicity, non-cancer—organics	CTUh	4.33 × 10^−8^	2.77 × 10^−8^
Ionizing radiation	kBq U-235 eq	3.0253	5.0298
Land use	Pt	201.9516	214.6499
Ozone depletion	kg CFC11 eq	1.90 × 10^−6^	3.86 × 10^−6^
Photochemical ozone formation	kg NMVOC eq	0.1124	0.1552
Resource use, fossils	MJ	403.6371	818.2176
Resource use, minerals and metals	kg Sb eq	0.0055	0.0001
Water use	m^3^ depriv.	11.0386	39.1682

**Table 5:** Comparative LCA for building façades in Poland: Damage category, damage assessment stage.

Damage Category	Unit	Ventilated Fascade with Rock Wool	ETICS with EPS-PL
Acidification	mol H+ eq	0.2776	0.4375
Climate change	kg CO_2_ eq	36.8156	42.5229
Ecotoxicity, freshwater	CTUe	98.436303	186.5544
Particulate matter	disease inc.	2.32 × 10^−6^	2.54 × 10^−6^
Eutrophication, marine	kg N eq	0.0368	0.0417
Eutrophication, freshwater	kg P eq	0.0176	0.0157
Eutrophication, terrestrial	mol N eq	0.4996	0.4173
Human toxicity, cancer	CTUh	6.47 × 10^−8^	5.78 × 10^−9^
Human toxicity, non-cancer	CTUh	1.07 × 10^−7^	2.93 × 10^−8^
Ionizing radiation	kBq U-235 eq	3.0253	5.0298
Land use	Pt	201.9516	214.6499
Ozone depletion	kg CFC11 eq	1.90 × 10^−6^	3.86 × 10^−6^
Photochemical ozone formation	kg NMVOC eq	0.1124	0.1552
Resource use, fossils	MJ	403.6371	818.2176
Resource use, minerals and metals	kg Sb eq	0.0055	0.0001
Water use	m^3^ depriv.	11.0386	39.1682

**Table 6:** Elements in the life cycle assessment for ventilated façades: damage category.

Damage Category	Unit	Fiber Cement Board with Lime	Mineral Wool with a Thick Veil, 15 cm Rock Wool	Aluminum Profiles	Consoles	The Remainder	Total
Acidification	mol H+ eq	0.0187	0.1619	0.0658	0.0176	0.013	0.2776
Climate change	kg CO_2_ eq	8.0991	16.6321	7.1364	1.9168	3.0310	36.8156
Ecotoxicity, freshwater	CTUe	11.1053	39.2803	33.7129	9.0552	5.2824	98.4363
Particulate matter	disease inc.	1.22 × 10^−7^	1.25 × 10^−6^	5.81 × 10^−7^	0	0	2.32 × 10^−6^
Eutrophication, marine	kg N eq	0.005	0.0156	0.0105	0.0028	0.0025	0.0368
Eutrophication, freshwater	kg P eq	0.0009	0.0051	0.0075	0.0020	0.0018	0.0176
Eutrophication, terrestrial	mol N eq	0.0594	0.2773	0.1065	0.0286	0.0276	0.4996
Human toxicity, cancer	CTUh	1.55 × 10^−9^	5.12 × 10^−8^	6.16 × 10^−9^	0	0	6.47 × 10^−8^
Human toxicity, non-cancer	CTUh	5.89 × 10^−8^	7.53 × 10^−9^	2.45 × 10^−8^	0	0	1.07 × 10^−7^
Ionizing radiation	kBq U-235 eq	0.3211	1.9180	0.5158	0.1385	0.1317	3.0253
Land use	Pt	6.9654	139.8213	37.8422	10.1643	7.1583	201.9517
Ozone depletion	kg CFC11 eq	4.15 × 10^−8^	1.13 × 10^−6^	5.09 × 10^−7^	0	0	1.90 × 10^−6^
Photochemical ozone formation	kg NMVOC eq	0.0164	0.0464	0.0314	0.0084	0.0096	0.1124
Resource use, fossils	MJ	34.0119	203.9107	82.41632	22.13688	61.16125	403.6371
Resource use, minerals and metals	kg Sb eq	8.14 × 10^−5^	4.61 × 10^−5^	0.0042	0.0011	0.0001	0.0055
Water use	m^3^ depriv.	0.5966	4.0063	4.0387	1.0847	1.3121	11.0386

**Table 7:** Share of individual elements in the life cycle assessment of the evaluated impact categories for ventilated façade system: damage category.

Damage Category	Fiber Cement Board with Lime	Mineral Wool with a Thick Veil, 15 cm Rock Wool	Aluminum Profiles	Consoles	The Remainder	Total
Acidification	6.74	58.34	23.72	6.37	4.83	100.00
Climate change	22.00	45.18	19.38	5.21	8.23	100.00
Ecotoxicity, freshwater	11.28	39.90	34.25	9.20	5.37	100.00
Particulate matter	5.26	53.88	25.04	0.00	0.00	100.00
Eutrophication, marine	14.37	42.45	28.56	7.67	6.95	100.00
Eutrophication, freshwater	5.34	29.43	42.95	11.54	10.73	100.00
Eutrophication, terrestrial	11.89	55.52	21.33	5.73	5.53	100.00
Human toxicity, cancer	2.40	79.13	9.52	0.00	0.00	100.00
Human toxicity, non-cancer	55.05	7.04	22.90	0.00	0.00	100.00
Ionizing radiation	10.62	63.40	17.05	4.58	4.35	100.00
Land use	3.45	69.24	18.74	5.03	3.54	100.00
Ozone depletion	2.18	59.47	26.79	0.00	0.00	100.00
Photochemical ozone formation	14.62	41.35	27.97	7.51	8.55	100.00
Resource use, fossils	8.43	50.52	20.42	5.48	15.15	100.00
Resource use, minerals and metals	1.46	0.83	75.87	20.39	1.47	100.00
Water use	5.41	36.29	36.59	9.83	11.89	100.00

**Table 8:** Elements in the life cycle assessment for ETICS: damage category.

Damage Category	Unit	EPS	Bonding Mortar	Base Coat	Fiberglass Mesh	Primer	Finish Coat	Total
Acidification	mol H+ eq	0.0486	0.0512	0.0512	0.0059	0.0146	0.2659	0.4375
Climate change	kg CO_2_ eq	11.6153	4.5844	4.5844	0.7836	1.0940	19.8610	42.5229
Ecotoxicity, freshwater	CTUe	6.2530	43.8055	43.8055	0.8448	4.7915	87.0539	186.5544
Particulate matter	disease inc.	5.08 × 10^−7^	2.81 × 10^−7^	2.81 × 10^−7^	4.96 × 10^−8^	7.43 × 10^−8^	1.35 × 10^−6^	2.54 × 10^−6^
Eutrophication, marine	kg N eq	0.0076	0.0048	0.0048	0.0011	0.0012	0.0220	0.0417
Eutrophication, freshwater	kg P eq	0.0014	0.0020	0.0020	0.0003	0.0005	0.0093	0.0157
Eutrophication, terrestrial	mol N eq	0.0818	0.0485	0.0485	0.0125	0.0117	0.2140	0.4173
Human toxicity, cancer	CTUh	5.54 × 10^−10^	1.30 × 10^−9^	1.30 × 10^−9^	5.97 × 10^−11^	1.37 × 10^−10^	2.44 × 10^−9^	5.78 × 10^−9^
Human toxicity, non-cancer	CTUh	5.21 × 10^−9^	3.48 × 10^−9^	3.48 × 10^−9^	2.33 × 10^−10^	8.81 × 10^−10^	1.60 × 10^−8^	2.93 × 10^−8^
Ionizing radiation	kBq U−235 eq	0.5658	0.6599	0.6599	0.1484	0.1564	2.8392	5.0298
Land use	Pt	16.16243	29.53729	29.53729	2.374006	7.150438	129.8885	214.65
Ozone depletion	kg CFC11 eq	2.96 × 10^−7^	5.55 × 10^−7^	5.55 × 10^−7^	7.42 × 10^−8^	1.24 × 10^−7^	2.26 × 10^−6^	3.86 × 10^−6^
Photochemical ozone formation	kg NMVOC eq	0.0494	0.0149	0.0149	0.0030	0.0038	0.0690	0.1552
Resource use, fossils	MJ	261.2882	79.2810	79.2810	13.3889	20.0912	364.8871	818.2177
Resource use, minerals and metals	kg Sb eq	2.12 × 10^−6^	2.28 × 10^−5^	2.28 × 10^−5^	4.48 × 10^−6^	6.10 × 10^−6^	0.000111	0.000169
Water use	m^3^ depriv.	8.1827	4.3320	4.3320	0.2472	1.2302	20.8439	39.16824

**Table 9:** Share of individual elements in the life cycle assessment of the evaluated impact categories for ETICS.

Impact Category	Unit	EPS	Bonding Mortar	Base Coat	Fiberglass Mesh	Primer	Finish Coat	Total
Acidification	mol H+ eq	11.11	11.71	11.71	1.35	3.34	60.78	100.00
Climate change	kg CO_2_ eq	27.32	10.78	10.78	1.84	2.57	46.71	100.00
Climate change—Biogenic	kg CO_2_ eq	45.05	7.67	7.67	0.71	2.03	36.86	100.00
Climate change—Fossil	kg CO_2_ eq	27.28	10.79	10.79	1.85	2.57	46.73	100.00
Climate change—Land use and LU change	kg CO_2_ eq	9.03	13.23	13.23	2.57	3.23	58.72	100.00
Ecotoxicity, freshwater—part 1	CTUe	3.22	22.12	22.12	0.42	2.72	49.40	100.00
Ecotoxicity, freshwater—part 2	CTUe	4.19	32.27	32.27	0.63	1.60	29.04	100.00
Ecotoxicity, freshwater—inorganics	CTUe	3.29	21.74	21.74	0.48	2.75	50.00	100.00
Ecotoxicity, freshwater—organics—p.1	CTUe	0.40	31.55	31.55	0.13	1.90	34.47	100.00
Ecotoxicity, freshwater—organics—p.2	CTUe	4.33	34.79	34.79	0.35	1.34	24.39	100.00
Particulate matter	disease inc.	19.97	11.05	11.05	1.95	2.92	53.05	100.00
Eutrophication, marine	kg N eq	18.31	11.60	11.60	2.80	2.91	52.79	100.00
Eutrophication, freshwater	kg P eq	9.45	12.91	12.91	1.99	3.28	59.45	100.00
Eutrophication, terrestrial	mol N eq	19.60	11.64	11.64	3.02	2.82	51.28	100.00
Human toxicity, cancer	CTUh	9.59	22.42	22.42	1.03	2.36	42.18	100.00
Human toxicity, cancer—inorganics	CTUh	8.54	19.58	19.58	1.11	2.67	48.52	100.00
Human toxicity, cancer—organics	CTUh	9.59	22.42	22.42	1.03	2.36	42.18	100.00
Human toxicity, non-cancer	CTUh	17.79	11.88	11.88	0.79	3.01	54.64	100.00
Human toxicity, non-cancer—inorganics	CTUh	8.70	11.74	11.74	3.06	3.38	61.40	100.00
Human toxicity, non-cancer—organics	CTUh	18.32	11.89	11.89	0.66	2.99	54.25	100.00
Ionizing radiation	kBq U-235 eq	11.25	13.12	13.12	2.95	3.11	56.45	100.00
Land use	Pt	7.53	13.76	13.76	1.11	3.33	60.51	100.00
Ozone depletion	kg CFC11 eq	7.66	14.35	14.35	1.92	3.22	58.50	100.00
Photochemical ozone formation	kg NMVOC eq	31.83	9.62	9.62	1.97	2.45	44.51	100.00
Resource use, fossils	MJ	31.93	9.69	9.69	1.64	2.46	44.60	100.00
Resource use, minerals and metals	kg Sb eq	1.26	13.49	13.49	2.65	3.61	65.49	100.00
Water use	m^3^ depriv.	20.89	11.06	11.06	0.63	3.14	53.22	100.00

## Data Availability

The original contributions presented in this study are included in the article/Supplementary Material. Further inquiries can be directed to the corresponding author.

## Supplementary Materials

The following supporting information can be downloaded at https://www.mdpi.com/article/10.3390/ma19040807/s1, Figure S1. Example of a facade that uses fiber-cement boards; Figure S2. Schematic cross-section of the ventilated facade system; Figure S3. Schematic cross-section of the ETICS system; Table S1. Elements in the life cycle assessment for ventilated facades—characterization stage; Table S2. Share of individual elements in the life cycle assessment of the evaluated impact categories for ventilated façade system—characterization stage, Table S3. Elements in the life cycle assessment for ETICS—characterization stage; Table S4. Share of individual elements in the life cycle assessment of the evaluated impact categories for ETICS—characterisation stage; Table S5. Uncertainty analysis for ventilated fascade—characterization; Table S6. Uncertainty analysis for ETICS—characterization.
