# Supplementary material for: Environmental Life Cycle Assessment of Selected Materials—Building Façades in Poland
Source: Materials (Basel). 2026 Feb 20;19(4):807. doi: 10.3390/ma19040807 (PMC12941522; doi:10.3390/ma19040807)
Supplement: Supplementary file 1 [file materials-19-00807-s001.zip › materials-4110620-supplementary.pdf]

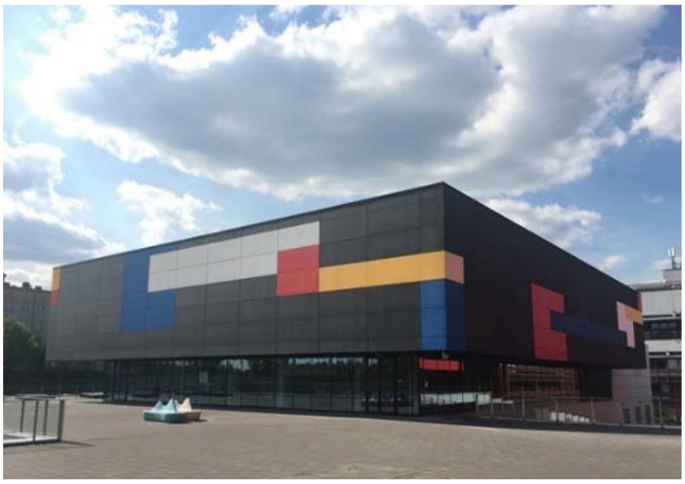

Figure S1. Example of a facade that uses fiber-cement boards

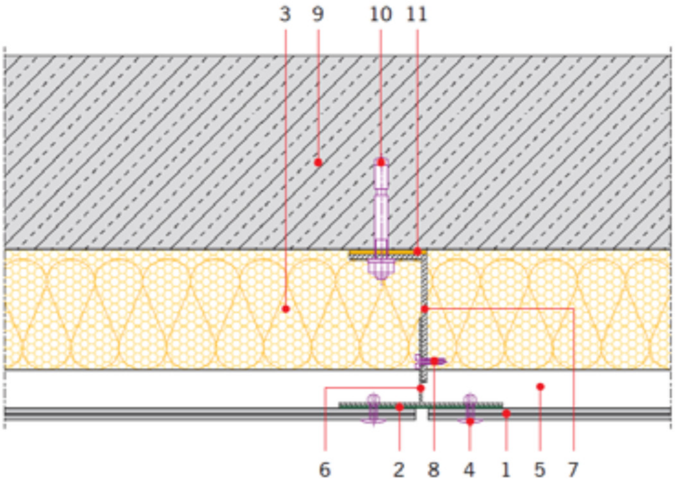

Figure S2. Schematic cross-section of the ventilated facade system
1 – facade cladding made of fiber-cement boards. 2 – EPDM tape. 3 – thermal insulation made of mineral wool with glass veil. 4 – board connector. 5 – ventilation gap. 6 – aluminum substructure (vertical load-bearing element). 7 – mounting console of vertical profiles. 8 – connector of aluminum elements. 9 – structural wall. 10 – anchor mounting the console. 11 – thermal insulation spacer

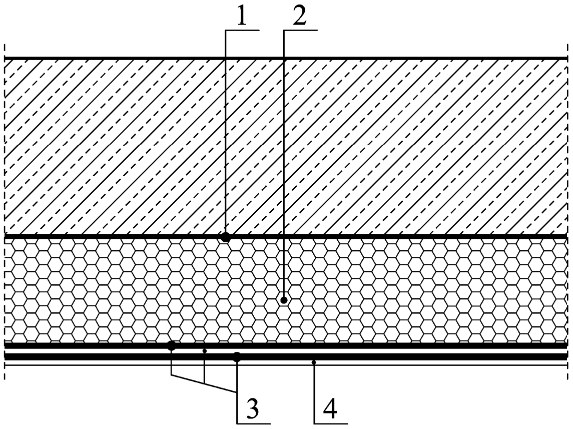

Figure S3. Schematic cross-section of the ETICS system.
1 – adhesive mortar. 2 – thermal insulation. 3 – reinforcing layer (mortar + fiberglass mesh). 4 – finishing facade plaster (thin-layer plaster); Thermal insulation can be additionally attached with mechanical connectors (pins)

Table S1. Elements in the life cycle assessment for ventilated facades – characterization stage

Impact category	Unit	fiber cement board with lime	Mineral wool with a thick veil. 15cm-rock wool	Aluminium profiles	Consoles	The remain elements	Total
Acidification	mol H+ eq	0.018707	0.161982	0.065855	0.017689	0.013408	0.277641
Climate change	kg CO2 eq	8.099129	16.63218	7.136454	1.916839	3.031007	36.81561
Climate change - Biogenic	kg CO2 eq	0.023193	0.014088	0.017022	0.004572	0.002831	0.061706
Climate change - Fossil	kg CO2 eq	8.074323	16.61097	7.03962	1.89083	3.026312	36.64206
Climate change - Land use and LU change	kg CO2 eq	0.001613	0.007117	0.079812	0.021437	0.001864	0.111842
Ecotoxicity. freshwater - part 1	CTUe	7.609165	34.62789	30.83572	8.282421	3.977223	85.33241
Ecotoxicity. freshwater - part 2	CTUe	3.496132	4.652484	2.8772	0.772811	1.305263	13.10389
Ecotoxicity. freshwater - inorganics	CTUe	10.53755	32.29373	26.20749	7.039285	4.724427	80.80248
Ecotoxicity. freshwater - organics - p.1	CTUe	0.127344	3.732615	5.473125	1.470072	0.099234	10.90239
Ecotoxicity. freshwater - organics - p.2	CTUe	0.440406	3.254024	2.032309	0.545875	0.458825	6.731437
Particulate matter	disease inc.	1.22E-07	1.25E-06	5.81E-07	1.56E-07	2.12E-07	2.32E-06
Eutrophication. marine	kg N eq	0.005294	0.015641	0.010523	0.002827	0.00256	0.036844
Eutrophication. freshwater	kg P eq	0.000943	0.005199	0.007586	0.002038	0.001896	0.017663
Eutrophication. terrestrial	mol N eq	0.059428	0.277391	0.106579	0.028627	0.027625	0.499649
Human toxicity. cancer	CTUh	1.55E-09	5.12E-08	6.16E-09	1.66E-09	4.1E-09	6.47E-08
Human toxicity. cancer - inorganics	CTUh	9.53E-10	8.87E-15	2.23E-16	5.98E-17	1.55E-10	1.11E-09
Human toxicity. cancer - organics	CTUh	5.98E-10	5.12E-08	6.16E-09	1.66E-09	3.95E-09	6.36E-08
Human toxicity. non-cancer	CTUh	5.89E-08	7.53E-09	2.45E-08	6.59E-09	9.55E-09	1.07E-07
Human toxicity. non-cancer - inorganics	CTUh	5.79E-08	1.96E-10	6.33E-10	1.7E-10	4.97E-09	6.38E-08
Human toxicity. non-cancer - organics	CTUh	1.04E-09	7.33E-09	2.39E-08	6.42E-09	4.58E-09	4.33E-08
Ionising radiation	kBq U-235 eq	0.321193	1.918032	0.515803	0.138544	0.131736	3.025307
Land use	Pt	6.965462	139.8213	37.84222	10.16435	7.158398	201.9517
Ozone depletion	kg CFC11 eq	4.15E-08	1.13E-06	5.09E-07	1.37E-07	8.41E-08	1.9E-06
Photochemical ozone formation	kg NMVOC eq	0.016438	0.046497	0.031459	0.00845	0.009614	0.112459
Resource use. fossils	MJ	34.01194	203.9107	82.41632	22.13688	61.16125	403.6371
Resource use. minerals and metals	kg Sb eq	8.14E-05	4.61E-05	0.004231	0.001137	8.15E-05	0.005577
Water use	m3 depriv.	0.59669	4.006304	4.038723	1.084794	1.312158	11.03867

Table S2. Share of individual elements in the life cycle assessment of the evaluated impact categories for ventilated façade system – characterization stage

Impact category	Fiber cement board with lime	Mineral wool with a thick veil. 15cm- rock wool	Aluminium profiles	Consoles	The remain elements	total
Acidification	6.74	58.34	23.72	6.37	4.83	100.00
Climate change	22.00	45.18	19.38	5.21	8.23	100.00
Climate change - Biogenic	37.59	22.83	27.59	7.41	4.59	100.00
Climate change - Fossil	22.04	45.33	19.21	5.16	8.26	100.00
Climate change - Land use and LU change	1.44	6.36	71.36	19.17	1.67	100.00
Ecotoxicity. freshwater - part 1	8.92	40.58	36.14	9.71	4.66	100.00
Ecotoxicity. freshwater - part 2	26.68	35.50	21.96	5.90	9.96	100.00
Ecotoxicity. freshwater - inorganics	13.04	39.97	32.43	8.71	5.85	100.00
Ecotoxicity. freshwater - organics - p.1	1.17	34.24	50.20	13.48	0.91	100.00
Ecotoxicity. freshwater - organics - p.2	6.54	48.34	30.19	8.11	6.82	100.00
Particulate matter	5.26	53.88	25.04	6.72	9.14	100.00
Eutrophication. marine	14.37	42.45	28.56	7.67	6.95	100.00
Eutrophication. freshwater	5.34	29.43	42.95	11.54	10.73	100.00
Eutrophication. terrestrial	11.89	55.52	21.33	5.73	5.53	100.00
Human toxicity. cancer	2.40	79.13	9.52	2.57	6.34	100.00
Human toxicity. cancer - inorganics	85.86	0.00	0.00	0.00	13.96	100.00
Human toxicity. cancer - organics	0.94	80.50	9.69	2.61	6.21	100.00
Human toxicity. non-cancer	55.05	7.04	22.90	6.16	8.93	100.00
Human toxicity. non-cancer - inorganics	90.75	0.31	0.99	0.27	7.79	100.00
Human toxicity. non-cancer - organics	2.40	16.93	55.20	14.83	10.58	100.00
Ionising radiation	10.62	63.40	17.05	4.58	4.35	100.00
Land use	3.45	69.24	18.74	5.03	3.54	100.00
Ozone depletion	2.18	59.47	26.79	7.21	4.43	100.00
Photochemical ozone formation	14.62	41.35	27.97	7.51	8.55	100.00
Resource use. fossils	8.43	50.52	20.42	5.48	15.15	100.00
Resource use. minerals and metals	1.46	0.83	75.87	20.39	1.46	100.00
Water use	5.41	36.29	36.59	9.83	11.89	100.00

Table S3. Elements in the life cycle assessment for ETICS – characterization stage

Impact category	Unit	EPS	Bonding mortar	Base coat	Fibre glass mesh	Primer	Finish coat	Total
Acidification	mol H+ eq	0.048626	0.051221	0.051221	0.005907	0.014636	0.265953	0.437564
Climate change	kg CO2 eq	11.61539	4.584408	4.584408	0.783658	1.09401	19.86104	42.52291
Climate change - Biogenic	kg CO2 eq	0.056566	0.00963	0.00963	0.000897	0.002548	0.04628	0.125552
Climate change - Fossil	kg CO2 eq	11.55625	4.571004	4.571004	0.782028	1.09054	19.798	42.36883
Climate change - Land use and LU change	kg CO2 eq	0.002576	0.003773	0.003773	0.000732	0.000923	0.016753	0.028531
Ecotoxicity. freshwater - part 1	CTUe	5.203568	35.72228	35.72228	0.685808	4.390801	79.77875	161.5035
Ecotoxicity. freshwater - part 2	CTUe	1.049531	8.083228	8.083228	0.159038	0.400735	7.275204	25.05097
Ecotoxicity. freshwater - inorganics	CTUe	5.286395	34.93946	34.93946	0.763492	4.42293	80.35947	160.7112
Ecotoxicity. freshwater - organics - p.1	CTUe	0.015598	1.223933	1.223933	0.004972	0.073566	1.337207	3.879208
Ecotoxicity. freshwater - organics - p.2	CTUe	0.951105	7.642109	7.642109	0.076383	0.295041	5.357273	21.96402
Particulate matter	disease inc.	5.08E-07	2.81E-07	2.81E-07	4.96E-08	7.43E-08	1.35E-06	2.54E-06
Eutrophication. marine	kg N eq	0.007645	0.004841	0.004841	0.001169	0.001214	0.022038	0.041747
Eutrophication. freshwater	kg P eq	0.001489	0.002035	0.002035	0.000313	0.000517	0.009369	0.015758
Eutrophication. terrestrial	mol N eq	0.081819	0.048563	0.048563	0.012584	0.011789	0.214025	0.417343
Human toxicity. cancer	CTUh	5.54E-10	1.30E-09	1.30E-09	5.97E-11	1.37E-10	2.44E-09	5.78E-09
Human toxicity. cancer - inorganics	CTUh	2.99E-16	6.86E-16	6.86E-16	3.88E-17	9.36E-17	1.70E-15	3.50E-15
Human toxicity. cancer - organics	CTUh	5.54E-10	1.30E-09	1.30E-09	5.97E-11	1.37E-10	2.44E-09	5.78E-09
Human toxicity. non-cancer	CTUh	5.21E-09	3.48E-09	3.48E-09	2.33E-10	8.81E-10	1.60E-08	2.93E-08
Human toxicity. non-cancer - inorganics	CTUh	1.40E-10	1.89E-10	1.89E-10	4.91E-11	5.43E-11	9.87E-10	1.61E-09
Human toxicity. non-cancer - organics	CTUh	5.07E-09	3.29E-09	3.29E-09	1.83E-10	8.27E-10	1.50E-08	2.77E-08
Ionising radiation	kBq U-235 eq	0.565819	0.659907	0.659907	0.148499	0.15644	2.839231	5.029802
Land use	Pt	16.16243	29.53729	29.53729	2.374006	7.150438	129.8885	214.65
Ozone depletion	kg CFC11 eq	2.96E-07	5.55E-07	5.55E-07	7.42E-08	1.24E-07	2.26E-06	3.86E-06
Photochemical ozone formation	kg NMVOC eq	0.049401	0.014937	0.014937	0.003058	0.003804	0.069081	0.155219
Resource use. fossils	MJ	261.2882	79.28109	79.28109	13.38897	20.09123	364.8871	818.2177
Resource use. minerals and metals	kg Sb eq	2.12E-06	2.28E-05	2.28E-05	4.48E-06	6.10E-06	0.000111	0.000169
Water use	m3 depriv.	8.182759	4.332032	4.332032	0.247258	1.230256	20.8439	39.16824

Table S4. Share of individual elements in the life cycle assessment of the evaluated impact categories for ETICS – characterisation stage

Impact category	Unit	EPS	Bonding mortar	Base coat	Fibre glass mesh	Primer	Finish coat	Total
Acidification	mol H+ eq	11.11	11.71	11.71	1.35	3.34	60.78	100.00
Climate change	kg CO2 eq	27.32	10.78	10.78	1.84	2.57	46.71	100.00
Climate change - Biogenic	kg CO2 eq	45.05	7.67	7.67	0.71	2.03	36.86	100.00
Climate change - Fossil	kg CO2 eq	27.28	10.79	10.79	1.85	2.57	46.73	100.00
Climate change - Land use and LU change	kg CO2 eq	9.03	13.23	13.23	2.57	3.23	58.72	100.00
Ecotoxicity. freshwater - part 1	CTUe	3.22	22.12	22.12	0.42	2.72	49.40	100.00
Ecotoxicity. freshwater - part 2	CTUe	4.19	32.27	32.27	0.63	1.60	29.04	100.00
Ecotoxicity. freshwater - inorganics	CTUe	3.29	21.74	21.74	0.48	2.75	50.00	100.00
Ecotoxicity. freshwater - organics - p.1	CTUe	0.40	31.55	31.55	0.13	1.90	34.47	100.00
Ecotoxicity. freshwater - organics - p.2	CTUe	4.33	34.79	34.79	0.35	1.34	24.39	100.00
Particulate matter	disease inc.	19.97	11.05	11.05	1.95	2.92	53.05	100.00
Eutrophication. marine	kg N eq	18.31	11.60	11.60	2.80	2.91	52.79	100.00
Eutrophication. freshwater	kg P eq	9.45	12.91	12.91	1.99	3.28	59.45	100.00
Eutrophication. terrestrial	mol N eq	19.60	11.64	11.64	3.02	2.82	51.28	100.00
Human toxicity. cancer	CTUh	9.59	22.42	22.42	1.03	2.36	42.18	100.00
Human toxicity. cancer - inorganics	CTUh	8.54	19.58	19.58	1.11	2.67	48.52	100.00
Human toxicity. cancer - organics	CTUh	9.59	22.42	22.42	1.03	2.36	42.18	100.00
Human toxicity. non-cancer	CTUh	17.79	11.88	11.88	0.79	3.01	54.64	100.00
Human toxicity. non-cancer - inorganics	CTUh	8.70	11.74	11.74	3.06	3.38	61.40	100.00
Human toxicity. non-cancer - organics	CTUh	18.32	11.89	11.89	0.66	2.99	54.25	100.00
Ionising radiation	kBq U-235 eq	11.25	13.12	13.12	2.95	3.11	56.45	100.00
Land use	Pt	7.53	13.76	13.76	1.11	3.33	60.51	100.00
Ozone depletion	kg CFC11 eq	7.66	14.35	14.35	1.92	3.22	58.50	100.00
Photochemical ozone formation	kg NMVOC eq	31.83	9.62	9.62	1.97	2.45	44.51	100.00
Resource use. fossils	MJ	31.93	9.69	9.69	1.64	2.46	44.60	100.00
Resource use. minerals and metals	kg Sb eq	1.26	13.49	13.49	2.65	3.61	65.49	100.00
Water use	m3 depriv.	20.89	11.06	11.06	0.63	3.14	53.22	100.00

Table S5. Uncertainty analysis for ventilated fascade – characterization

Impact category	Unit	Mean	Median	SD	CV	2.50%	97.50%	SEM
Acidification	mol H+ eq	0.2779423	0.276868	0.020368	7.328307	0.240809	0.321325	0.000644107
Climate change	kg CO2 eq	36.841163	36.66618	2.615129	7.098389	32.47566	42.48528	0.082697645
Climate change - Biogenic	kg CO2 eq	0.061506096	0.061124	0.004947	8.042414	0.052848	0.07307	0.000156424
Climate change - Fossil	kg CO2 eq	36.668075	36.47906	2.613331	7.126993	32.30596	42.31421	0.082640789
Climate change - Land use and LU change	kg CO2 eq	0.11158235	0.111926	0.012381	11.09621	0.0869	0.135772	0.000391535
Ecotoxicity. freshwater - inorganics	CTUe	80.779205	82.20983	46.25843	57.26527	-13.2073	172.7736	1.4628201
Ecotoxicity. freshwater - organics - p.1	CTUe	10.834678	10.59978	1.726924	15.93886	8.113678	14.71279	0.054610123
Ecotoxicity. freshwater - organics - p.2	CTUe	6.7854822	6.523692	1.398403	20.60875	4.692873	10.21341	0.044221382
Ecotoxicity. freshwater - part 1	CTUe	85.251957	86.90112	46.37027	54.39203	-8.23316	176.7539	1.4663566
Ecotoxicity. freshwater - part 2	CTUe	13.147407	12.75107	2.264564	17.22442	9.95526	19.00698	0.071611803
Eutrophication. freshwater	kg P eq	0.017641013	0.015729	0.007647	43.34956	0.009705	0.034929	0.000241829
Eutrophication. marine	kg N eq	0.036863925	0.036668	0.002637	7.15371	0.03226	0.042279	8.34E-05
Eutrophication. terrestrial	mol N eq	0.50081764	0.501519	0.04343	8.671874	0.423007	0.59098	0.001373386
Human toxicity. cancer	CTUh	6.41E-08	6.38E-08	1.90E-08	29.61318	2.76E-08	1.00E-07	6.00E-10
Human toxicity. cancer - inorganics	CTUh	9.50E-10	7.48E-10	1.42E-08	1498.059	-2.69E-08	2.79E-08	4.50E-10
Human toxicity. cancer - organics	CTUh	6.31E-08	6.11E-08	1.27E-08	20.12435	4.28E-08	9.19E-08	4.02E-10
Human toxicity. non-cancer	CTUh	9.86E-08	1.06E-07	3.25E-06	3298.875	-6.13E-06	6.45E-06	1.03E-07
Human toxicity. non-cancer - inorganics	CTUh	5.55E-08	7.28E-08	3.25E-06	5859.689	-6.16E-06	6.40E-06	1.03E-07
Human toxicity. non-cancer - organics	CTUh	4.31E-08	4.13E-08	9.78E-09	22.69225	2.95E-08	6.62E-08	3.09E-10
Ionising radiation	kBq U-235 eq	2.9958134	2.339981	2.535698	84.64138	1.217754	8.834309	0.080185802
Land use	Pt	202.16442	189.9123	59.43203	29.39787	123.7189	353.2363	1.8794058
Ozone depletion	kg CFC11 eq	1.92E-06	1.86E-06	3.78E-07	19.71747	1.39E-06	2.79E-06	1.20E-08
Particulate matter	disease inc.	2.34E-06	2.26E-06	4.53E-07	19.39795	1.73E-06	3.41E-06	1.43E-08
Photochemical ozone formation	kg NMVOC eq	0.11246616	0.112003	0.007863	6.991195	0.099379	0.129735	0.000248641
Resource use. fossils	MJ	403.25004	401.2204	28.57708	7.086689	353.6799	464.0144	0.90368648
Resource use. minerals and metals	kg Sb eq	0.005591564	0.005535	0.000775	13.8526	0.00427	0.00736	2.45E-05
Water use	m3 depriv.	2.4338061	40.24553	328.7894	13509.27	-719.326	560.2848	10.397235

Table S6. Uncertainty analysis for ETICS– characterization

Impact category	Unit	Mean	Median	SD	CV	2.50%	97.50%	SEM
Acidification	mol H+ eq	0.436033	0.423896	0.096037	22.02527	0.297071	0.660522	0.003037
Climate change	kg CO2 eq	42.82819	42.52775	4.216874	9.846023	35.10474	51.90974	0.133349
Climate change - Biogenic	kg CO2 eq	0.127144	0.122822	0.026633	20.947	0.088132	0.188954	0.000842
Climate change - Fossil	kg CO2 eq	42.67243	42.3856	4.200958	9.844667	34.98396	51.71601	0.132846
Climate change - Land use and LU change	kg CO2 eq	0.028623	0.028195	0.007005	24.473	0.016093	0.042212	0.000222
Ecotoxicity. freshwater - inorganics	CTUe	160.2631	158.0243	24.22574	15.11623	116.5629	210.5786	0.766085
Ecotoxicity. freshwater - organics - p.1	CTUe	3.867704	3.751212	0.853958	22.07919	2.565032	5.793	0.027005
Ecotoxicity. freshwater - organics - p.2	CTUe	21.44863	19.74242	8.36102	38.9816	10.84456	43.67798	0.264399
Ecotoxicity. freshwater - part 1	CTUe	161.0247	158.5627	24.08746	14.95886	117.5883	211.9335	0.761712
Ecotoxicity. freshwater - part 2	CTUe	24.55472	22.90225	8.411649	34.25675	13.86534	46.35072	0.266
Eutrophication. freshwater	kg P eq	0.015783	0.014251	0.007461	47.27056	0.006938	0.033022	0.000236
Eutrophication. marine	kg N eq	0.042119	0.041817	0.004534	10.7654	0.033967	0.051458	0.000143
Eutrophication. terrestrial	mol N eq	0.421259	0.418414	0.045237	10.73855	0.340437	0.51555	0.001431
Human toxicity. cancer	CTUh	5.82E-09	5.73E-09	9.05E-10	15.55318	4.40E-09	7.91E-09	2.86E-11
Human toxicity. cancer - inorganics	CTUh	3.53E-15	3.45E-15	5.44E-16	15.41502	2.73E-15	4.73E-15	1.72E-17
Human toxicity. cancer - organics	CTUh	5.82E-09	5.73E-09	9.05E-10	15.55318	4.40E-09	7.91E-09	2.86E-11
Human toxicity. non-cancer	CTUh	2.94E-08	2.85E-08	6.11E-09	20.77731	2.05E-08	4.50E-08	1.93E-10
Human toxicity. non-cancer - inorganics	CTUh	1.63E-09	1.44E-09	7.75E-10	47.54317	8.23E-10	3.86E-09	2.45E-11
Human toxicity. non-cancer - organics	CTUh	2.78E-08	2.68E-08	5.95E-09	21.42607	1.91E-08	4.29E-08	1.88E-10
Ionising radiation	kBq U-235 eq	5.074629	3.781656	4.327544	85.27804	1.972591	16.36076	0.136849
Land use	Pt	215.1393	212.6991	41.15134	19.12776	141.1389	303.5013	1.30132
Ozone depletion	kg CFC11 eq	3.91E-06	3.77E-06	9.26E-07	23.67962	2.59E-06	6.22E-06	2.93E-08
Particulate matter	disease inc.	2.56E-06	2.53E-06	3.45E-07	13.48956	1.95E-06	3.29E-06	1.09E-08
Photochemical ozone formation	kg NMVOC eq	0.156078	0.154742	0.019401	12.43058	0.122098	0.199053	0.000614
Resource use. fossils	MJ	823.3823	814.8157	88.24835	10.71779	669.2791	1011.973	2.790658
Resource use. minerals and metals	kg Sb eq	0.00017	0.000148	8.62E-05	50.65679	7.21E-05	0.000381	2.73E-06
Water use	m3 depriv.	34.12324	46.71158	206.1655	604.1792	-390.299	404.694	6.519527
